# Cognitive Fluency in L2: The Effect of Automatic and Controlled Lexical Processing on Speech Rate

**DOI:** 10.1007/s10936-024-10099-0

**Published:** 2024-08-20

**Authors:** Sanna Olkkonen, Patrick Snellings, Outi Veivo, Pekka Lintunen

**Affiliations:** 1 Department of English, University of Turku, Turku, Finland; 2 Department of Developmental Psychology, University of Amsterdam, Amsterdam, The Netherlands; 3 Rudolf Berlin Center, Amsterdam, The Netherlands; 4 Department of French, University of Turku, Turku, Finland

**Keywords:** Cognitive fluency, Speech rate, Oral fluency, Second language acquisition

## Abstract

The fluency of second language (L2) speech can be influenced by L2 proficiency, but also by differences in the efficiency of cognitive operations and personal speaking styles. The nature of cognitive fluency is still, however, little understood. Therefore, we studied the cognitive fluency of Finnish advanced students of English (N = 64) to understand how the efficiency of cognitive processing influences speech rate. Cognitive fluency was operationalised as automaticity of lexical access (measured by rapid word recognition) and attention control (measured by the Stroop task). The tasks were conducted in both L1 (Finnish) and L2 (English) to examine the (dis)similarity of processing in the two languages. Speech rate in a monologue task was used as the dependent measure of speaking performance. The results showed that after controlling for the L1 speech rate and L1 cognitive fluency, the L2 attention control measures explained a small amount of additional variance in L2 speech rate. These results are discussed in relation to the cognitive fluency framework and general speaking proficiency research.

Using a second language (L2) fluently is the goal of many language learners, and understanding which factors determine fluent language use is crucial for both assessment and teaching (see, e.g., Lintunen et al., 2020). This applies especially to oral fluency, which is prone to subjective evaluation if not clearly defined and instructed (e.g., De Jong, 2018). An influential framework for studying L2 fluency by Segalowitz (2010, 2016) has three dimensions: the subjective judgments on fluency (*perceived fluency*), the temporal features of speech (*utterance fluency*), and the efficiency of cognitive processes underlying speech production (*cognitive fluency*). These dimensions are linked: for instance, utterance fluency influences how oral fluency is perceived, and cognitive fluency influences how fluently utterances are produced (Segalowitz & Freed, 2004). However, the exact nature of the relationships between these different dimensions is still unclear, and especially the role of cognitive fluency, which remains quite elusive even as a construct (De Jong, 2018). The present paper, therefore, aims to fill this gap by examining the relationship between cognitive fluency and an aspect of utterance fluency (i.e., speech rate).

Cognitive fluency was approached from the viewpoint of allocation and efficiency of processing resources. The efficiency of cognitive processing was measured by lexical access (see Just & Carpenter, 1992; LaBerge & Samuels, 1974; Snellings et al., 2002), which is one the areas of L2 skills that remains slower compared to L1 even at a very advanced level (Levelt, 1989; Segalowitz, 2010). Therefore, lexical access was measured in both L1 (Finnish) and L2 (English), to be able to assess the discrepancy of processing between languages. The interest was especially in, on one hand, how automatic lexical access was for the advanced learner participants, and, on the other hand, how efficiently they could control their output. To study the effect of these processes on L2 speaking performance, a monologue task was conducted. Monologues were performed in L1 as well, to take into account the effect of personal speaking style. Speech rate was used as a measure of speaking performance, as it is a widely used composite measure that covers both speed of production and breakdown fluency (i.e., pausing; see, e.g., Lennon, 1990). Encompassing both speed and breakdown fluency was deemed important as the speed of production as well as aspects of pausing are highly correlated between L1 and L2 speech (e.g., Derwing et al., 2009; Duran-Karaoz & Tavakoli, 2020). We start by reviewing studies on cognitive fluency and presenting a framework of cognitive resources, which can influence speaking performance.

## Conceptualising Cognitive Fluency

Individual cognitive factors affecting oral fluency reflect “the efficiency of operation of the underlying processes” of speech production (Segalowitz, 2010: 165). Cognitive fluency, thus, includes the processes and abilities that help or hinder speakers’ fluent performance and are reflected in utterance fluency, for instance, in speech rate. The existing research has aimed to establish links between aspects of cognitive fluency, operationalised as the range of linguistic resources available (e.g., vocabulary size) and processing speed (e.g., lexical or grammatical retrieval speed), and specific utterance fluency features, such as mean syllable duration and speech rate (De Jong et al., 2013; Kahng, 2020; Suzuki & Kormos, 2022). In general, studies have consistently shown a relationship between processing speed, and both speed and breakdown fluency, whereas the use of linguistic resources may be more dependent on the demands of the task (Suzuki & Kormos, 2022).

In addition to the linguistic resources and processing efficiency aspects, Segalowitz’s (2010) cognitive fluency model includes the component of *attention control*, labelled as one of the most important mechanisms differentiating expert language users from beginners (Segalowitz & Frenkiel-Fishman, 2005). There is, however, a notable gap in L2 speech fluency research including attention control as a variable of cognitive fluency (with the noteworthy exception of Segalowitz & Freed, 2004). Controlling attention requires cognitive resources (Just & Carpenter, 1992), and these resources are more stretched for learners of any skill (e.g., Towell & Dewaele, 2005). Resources for attention control are freed up by the automatization of processes (Segalowitz, 2010), and the interplay of automaticity and attention control is considered the basis of any fluent action, for example, reading fluency and comprehension (Kieffer & Christodoulou, 2020; LaBerge & Samuels, 1974). Processes develop from controlled to more automatic with training (Abutalebi & Green, 2007; Segalowitz, 2010: 142–3), but, for example in the case of L2, their distribution is still quite different even at a very advanced stage (e.g., in word naming, see Plat et al., 2018). There is some evidence, however, that, with more automatic processing, the L2 speech processes approach the L1 processing (Duran-Karaoz & Tavakoli, 2020; Gao & Sun, 2023). Therefore, despite the general results of the contribution of automaticity and attention control to L2 skills, little is still understood of their relationship to L2 speaking performance, which the present study will address. Next, previous findings regarding automatic and controlled processing that have bearing on L2 speaking are discussed.

## Automaticity and L2 Speaking

Automaticity means accurate, fast, and stable performance that is, however, also ballistic in nature (e.g., Plat et al., 2018; Segalowitz, 2010, 2016). Automatization concerns especially lower-level processes, such as lexical access, which is one of the aspects in which L1 and L2 performance differ greatly (Levelt, 1989). Efficient lexical access is considered a central aspect of cognitive fluency (Segalowitz, 2010: 75–6; Segalowitz & Freed, 2004), but more research is needed on how advanced L2 learners differ in their levels of automaticity of lexical access and the effect this has on their speech performance.

Tasks aimed at measuring the automaticity of language processing should be cognitively simple, that is, not pose demands on planning or conceptualisation (e.g., Felker et al., 2019), and have little overlap with the speaking task itself (Segalowitz, 2016). One option, therefore, is *rapid word recognition* (see, e.g., Leinonen et al., 2001; Roembke et al., 2019). In the task, the participants name words aloud after a very short exposure, on the threshold of reading (to tap sight-word reading; see Ehri, 1991). The assumption is that with little time for decoding the word, the readers have to rely on the automaticity of word recognition (e.g., Leinonen et al., 2001; Zeguers et al., 2014). Therefore, with short exposure time and long enough words, it is possible to tap into the differences between the level of automaticity of L1 and L2 lexical access even for advanced L2 users.

## Attention Control and L2 Speaking

The influence of attention control on L2 use has been studied in relation to different language functions, such as focusing and language selection (Segalowitz, 2010: 93). Attention control can also be examined within the framework of *executive functions* (EFs), neuropsychological mechanisms that control and direct mental processes and actions. The most prominent way to classify the EFs is a division into *updating* information and monitoring, *inhibition* of distractions, and *switching* from one set to another (Miyake et al., 2000). Shifting and selection of attention are also included in Levelt’s (1989) model as controlled parts of speech production. Nevertheless, to the best of our knowledge, the influence of EFs on speaking performance has mainly been discussed in relation to diagnostic speech difficulties in L1 environment (see, e.g., in Parkinson’s: Hedman et al., 2022; in ADHD: Engelhardt et al., 2010; for the influence on general speech production, see, e.g., Linck et al., 2020). In contrast to automaticity, attention control seems not to be directly connected to L2 skills. On the one hand, monitoring depends on the amount of available resources (e.g., Levelt, 1989), L2 proficiency (Kormos, 2000; Olkkonen, 2017), and individual cognitive and style differences (Zuniga & Simard, 2019). On the other hand, it is more difficult to inhibit the activation of the language one is more proficient in, and more cognitive resources are needed to suppress interference (Meuter & Allport, 1999). These findings suggest that, for example, repairs and interference-induced errors may be more prevalent in one’s more advanced language. In fact, Segalowitz and Freed (2004) found that the efficiency of attention control was negatively related to L2 speech rate, which was explained as the better ability to monitor one's own speech. Therefore, more information is needed on how monitoring influences L2 speech (see, Segalowitz, 2016; Kahng, 2020; Suzuki & Kormos, 2022), especially of advanced L2 users.

Inhibition efficiency is often studied with a Stroop task (Stroop, 1935; see review in MacLeod, 1991). In one typical version of the task, colour words and neutral words are written in different colour inks, and the participant is asked to name the ink, ignoring the word. Reaction times between neutral, congruent (colour word and ink match), and incongruent (colour word and ink do not match, e.g., the word *red* written in green) conditions are compared. The more automatic language processing is, the more *interference* it induces in the incongruent situation, and if measured multilingually, the amount of cross-linguistic interference also serves as an indicator of the level of automaticity of word activation (e.g., Heidlmayr et al., 2014; MacLeod, 1991). The interference effect has, consequently, been found only for very advanced language skills (L1 or L2, but not for L3; Marian et al., 2013). More proficient languages have also produced a stronger *facilitation* effect in the congruent situation, although only for certain language combinations (Van Heuven et al., 2011). However, the influence these effects have on L2 speech rate has not been investigated, and understanding this connection may offer information on the relationship between L1 and L2 processing during speech for advanced L2 users.

To summarise, automaticity and cognitive control are supposed to be somewhat complementary skills, with automaticity releasing resources for the controlled processes. Examining how these differ between L1 and L2 may help to disentangle the influence of cognitive processing skills on L2 speaking. Especially, differentiating between general cognitive efficiency (as measured by L1 tasks) and L2-specific efficiency (L2 tasks) can shed light on how the different profiles in automaticity and attention control are potentially reflected in speaking performance, for instance, in speech rate.

## Measurement of L2 Speaking Performance

For speaking performance, one of the most widely used measures is speech rate. Speech rate measures the number of syllables or words produced in a certain time, often including pausing and/or disfluencies. It is, therefore, a global composite measure, which encompasses both speed and breakdown (i.e., pausing aspects) of speaking performance. Speech rate has been shown to correlate highly with L2 proficiency (Duran-Karaoz & Tavakoli, 2020; Iwashita et al., 2008), and is deemed one of the main components in fluency ratings, as well as distinguishing between advanced and intermediate students (e.g., Kormos & Dénes, 2004). Therefore, its relationship to L2 speaking performance is well established; however, studies that have examined the link between speech rate and cognitive processing are few to date (with the exception of Segalowitz & Freed, 2004).

Strong correlations have been found, however, also between L1 and L2 speech rates (Derwing et al., 2009; Gagné et al., 2022; Peltonen, 2020). This points to a more individual speaking style component in the speech rate, as also pausing behaviour is found to be linked between L1 and L2 (Gao & Sun, 2023; Towell & Dewaele, 2005). In addition, disfluencies usually do not correlate with proficiency (e.g., Kormos & Dénes, 2004), and the use of repairs in speech is influenced by individual preferences to an extent (Duran-Karaoz & Tavakoli, 2020; Kahng, 2014; Olkkonen, 2017). Taken together, these results highlight the need to account for the variance in general speaking style with the measurement of L1 speech rate, to control for the speed, breakdown and disfluencies that are shared between the languages, to tease out the L2-specific components of cognitive fluency.^1^ Additionally, regarding the connections of speech rate to both proficiency level and L1 speaking style, it is possible that the latter (i.e., connection to L1 speech rate) is more pronounced for the more proficient L2 speakers (see e.g., Duran-Karaoz & Tavakoli, 2020; Olkkonen et al., 2024). As most L2 fluency studies have been conducted with beginning to intermediate level learners (Derwing et al., 2009; Duran-Karaoz & Tavakoli, 2020; Gagné et al., 2022; Kahng, 2020), in the current study we concentrated on a group of advanced language users to examine the extent to which L1 measures (both speech rate and cognitive fluency) are able to explain L2 speech.

## The Present Study

The aim of the present study is to extend previous research on L2 cognitive fluency by examining the relationship of the efficiency of cognitive processing on L2 speech rate. Based on the theory of cognitive fluency as an interplay of automatic and controlled processing (LaBerge & Samuels, 1974; Segalowitz, 2010), cognitive fluency was measured with two lexical access tasks: rapid word recognition (automaticity) and the Stroop task (attention control). Better automaticity was hypothesised to relate to more fluent and proficient language skills, so that more fluent speakers also have more automatic access to their lexicon than less fluent ones, which could lead to a higher speech rate (see also, e.g., Duran-Karaoz & Tavakoli, 2020). On the other hand, higher automaticity can also lead to more difficulties in attention control (e.g., inhibition) and therefore, to more repairs (Plat et al., 2018; Segalowitz & Freed, 2004). Therefore, the number of repairs in the Stroop task was measured, to tap into self-monitoring processes. Conducting the low-level tasks in cognitive processing in both L1 and L2 allowed us to compare possible differences in efficiency of utilising cognitive resources (see Segalowitz, 2010). We measured the participants’ speech rate in a short monologue task. Controlling for the L1 speech rate, furthermore, was deemed to reveal specific differences between L1 and L2 processing in speech production, rather than in speaking style (e.g., pausing behaviour).

The specific research questions were:

- *RQ 1* To what extent are automatic and controlled lexical access in L1 and L2 related to each other for advanced L2 users?
- *RQ 2* To what extent can the automatic and controlled lexical access measures explain variance in speech rate for advanced L2 users, after controlling for speech rate in L1?

## Methods

### Participants

The participants were university language students (N = 64) at a Finnish university (mean age = 22.7, standard deviation = 5.5). All participants were native Finnish speakers with advanced English skills (number of study years M = 10.2, SD = 1.3). Their proficiency in the L2 was measured with the LexTale vocabulary test (Lemhöfer & Broersma, 2012), a non-speeded English lexical decision task aimed specifically at high-proficiency users. All participants studied languages either as their major or minor subject and were at least on the B2 level (LexTale score M = 84.9, SD = 8.7), with 70.3% on the C1/C2 level in the Common European Framework of Reference scale (CEFR; Council of Europe, 2001). Signed informed consents were gathered from all participants, and the data were processed according to University of Turku ethical and GDPR guidelines. The students participated in the test battery in a group setting in a university language studio and computer lab as part of their course work (see Table 1 for overview of the measures). All participants had normal or corrected-to-normal vision, and no language-related problems. Colour blindness was, however, an exclusion criterion because of the Stroop task.

**Table 1:** Fluency dimensions included in the study

Fluency dimension	Tasks	Language	Object of measure
1. Cognitive fluency			
Automaticity	Rapid words	L1 & L2	Rapid holistic word recognition
Attention control	Stroop task	L1 & L2	Attention control in naming colours Inhibition: interference and facilitation Monitoring: number of repairs
2. Oral fluency	Monologue (comic strip)	L1 & L2	Speech rate (number of syllables/speaking time)

### Fluency Tasks

#### Cognitive Fluency

The cognitive fluency tasks (Rapid words and Stroop task) were administered using jsPsych 6.3 (De Leeuw, 2015), a JavaScript framework for web-based behavioural experiments. The tasks were counterbalanced in language blocks, so the participants conducted both tests first either in the L1 or L2. The whole set of tasks lasted approximately 10 min. The stimuli were presented in monospace 64-point Consolas lowercase font.

##### Rapid Words

The participants were asked to name aloud 30 written words per language, flashed on the screen for 50 ms in a fixed order, becoming progressively more difficult in both length and frequency (see Appendix 1 for complete word lists). The participants were instructed to say the words aloud as fast and accurately as they could, and were encouraged to guess if not sure. The task started with a fixation point and four training words. Immediately after each word, a mask the length of the longest word (string of #####) was presented on the screen for 500 ms to erase the afterimage, and ISI was 2000 ms to have enough time not to put pressure on production. The tasks were first pilot-tested with five matched participants, after which some items were changed for a better suit (high-frequency words were at ceiling in both languages) and re-piloted with an additional group of five participants.

The exposure time was set low as most orthographic information needed for word recognition can be accessed already in less than 60 ms in L1 (Leinonen et al., 2001; Zeguers et al., 2018), and based on piloting, this was possible in L2 as well. For the task to measure automaticity rather than decoding processes, a backward mask has been shown to be an especially good tool to end the bottom-up visual processing (Roembke et al., 2019). The length (range 8–13 letters; Finnish mean 10.2 letters, English mean 9.8 letters) and frequency (mid- to low-frequency word families from 3000 to 9000 + words; Finnish frequencies: Parole database, 2007; English: Nation, 2017) of the L1 and L2 items were matched as closely as possible. The English words were slightly shorter, as L2 is suggested to be more sensitive to length effects (see, e.g., de Groot et al., 2002), and the limit for L2 perceptual span is suggested to be around eight characters to the right (Leung et al., 2014). The most difficult words were conjugated words in Finnish and multipart words in English. Neutrality and low imageability were favoured, and cognate and compound words were excluded. The answers were scored for their accuracy, and points were given on partial recognition (such as reactivate > react, reactive; 1 for correct, 0.5 for a correct base word and 0.25 for a correct part). The accuracy of the responses was evaluated by the first author and a research assistant, and the interrater reliability of the accuracy scores was 0.87 (Cohen’s Kappa).

##### Stroop Task

The task consisted of neutral and colour words, written in different colour inks, and the participants were instructed to name the colour of the ink aloud as quickly and as accurately as they could. The task consisted of four colour words in Finnish and in English (*punainen/red, sininen/blue, keltainen/yellow, vihreä/green*) and three neutral words (*ovi/door, ikkuna/window, tuoli/chair*), printed in red, blue, yellow or green ink on grey background. There were 72 items in both L1 and L2 trials, with each colour presented 12 times in each of the six conditions (neutral, congruent, and incongruent in L1 and L2; see Appendix 2 for the list of items used). In the congruent condition, the word was printed in the corresponding colour (*red/punainen* in red); in the incongruent condition, in a different colour (*red/punainen* in green); and in the neutral condition, a neutral word in one of the four colours (*door/ovi* in red). Moreover, the stimuli words could be either in L1 or L2 (mixed design), but the answering language was blocked (either L1 or L2 first). This design was chosen to obtain cross-linguistic interference and facilitation variables, to be able to compare the automatic activation of each language in the situation where it was irrelevant. The neutral words were chosen so as not to share initial letters with the colour words in either language (see Heidlmayr et al., 2014).

The order of the stimuli was pseudorandomized using lists created with Mix program (van Casteren & Davis, 2006), with the following constraints (based on Heidlmayr et al., 2014): no more than two stimuli of the same experimental condition were presented in succession, no word or print colour was repeated immediately, and the first stimulus was always a neutral one. Additionally, a maximum of three consecutive stimuli in the same language was allowed. The participants were randomly allocated to one of the possible lists per language. The trial began with 8 training items, with the correct answers provided. After a fixation sign (+) for 500 ms, the stimulus word appeared for 1200 ms, and then a blank screen for 500 ms. Naming reaction times (RTs) from the beginning of the correct answer were measured using Praat software (Boersma & Weenink, 2022) manually by research assistants, and 10% were double-checked by the first author. Based on the mean RTs in each condition, *Interference* effects (mean RTs in Incongruent condition minus RTs in Neutral condition) and *Facilitation* effects (RTs in Neutral condition minus RTs in Congruent condition) were calculated for each language and cross-language conditions separately. Thus, a higher score (larger difference in RTs between the conditions) marks more interference and more facilitation. Wrong answers and late responses (over 2100 ms) were excluded (0.6% of all answers), but the number of repairs was counted. In the Stroop task, the repairs are mostly false starts (rejected or cut-off sounds, words, or longer utterances) or replacements (words replaced with another one, without additional modifications) (following Foster & Skehan, 1999). The repairs were calculated to obtain, in addition to interference, an index of monitoring in attention control. There was some variance in the audio length due to the chosen platform (60 ms deviation), but this was found not to affect the RTs of different conditions systematically.

#### Speaking Task

Speaking performance was measured with a monologue task of telling a short story based on two comparable cartoon strips of six pictures, with a planning time of two minutes, and instructions to describe the storyline in their own words (for the procedure, see Peltonen, 2020; see also Lennon, 1990). The monologues were conducted in both L1 and L2, with the order of the language and the prompt pictures counterbalanced between the participants. The monologues were transcribed, annotated, and double-checked by research assistants under the supervision of the research team, using Praat software (Boersma & Weenink, 2022). The task covers the complete process of speech production from conceptualisation to articulation (Levelt, 1989; see also, Towell & Dewaele, 2005), and is thought, therefore, to offer a good sample of an individual’s speaking style as a whole. In the current analysis, speech rate was used as a measure of speaking performance. Speech rate (number of syllables/total speaking time × 60, to standardise per minute) included both pausing time and disfluencies (filled pauses, hesitations).

## Results

The statistical analyses were performed with SPSS (version 27). Descriptive statistics for the six cognitive fluency measures in both languages as well as the speech rates in L1 and L2 (means (M) and standard deviations (SD)) are presented in Table 2. The participants were, on average, more accurate in the rapid words in L1 than in L2 (accuracy percentage 77% for the L1 and 51% for the L2). Their speech rate was also higher in L1 than in L2 (228.4 and 153.9 syllables per minute in L1 and L2, respectively).

**Table 2:** Descriptive statistics of rapid word task, Stroop task and monologue task

Task	L1 M (SD)	L2 M (SD)
Rapid words (accuracy %)	77.3 (17.6)	50.8 (20.1)
*Stroop task conditions (RTs in ms)*		
Interference effect	118.1 (97.1)	79.9 (83.4)
Facilitation effect	41.5 (78.6)	84.3 (96.2)
Cross-language Interference	L1 > L2: 107.8 (91.2)	L2 > L1: 88.0 (73.7)
Cross-language Facilitation	44.9 (81.3)	20.5 (65.9)
Stroop repairs (number)	1.4 (1.8)	1.0 (1.4)
*Speech monologue*		
Length (in seconds)	63.7 (19.7)	68.3 (24.4)
Syllables (mean)	239.5 (78.6)	174.2 (68.7)
Speech rate (syllables/total time*60)	228.4 (41.9)	153.9 (28.0)
Min	123	104
Max	316	227

Regarding RQ 1, to examine the connections between the cognitive fluency measures in L1 and L2 (automatic and controlled lexical access), Pearson correlations were calculated (see Table 3 for the results). For accuracy in Rapid words (automaticity), there was a high correlation between the L1 and L2 scores (*r* = 0.59), which was significant after the Bonferroni correction. The interference and facilitation effects in the Stroop task had only low correlations with each other; however, the number of repairs in the task had a high and significant correlation across the languages (*r* = 0.70).

**Table 3:** Pearson correlations between L1 and L2 cognitive measures

Measure	L1–L2 (FI–EN)
Rapid words	.59**
Interference (Stroop)	.19
Facilitation (Stroop)	.25
Repairs (Stroop)	.70**

** *p* < 0.05 level (after Bonferroni correction)

To answer RQ 2, whether the cognitive fluency measures explain variance in the L2 speech rate, a regression analysis was performed. The regression analysis was conducted in two phases: first, a linear regression model was performed with L1 speech rate and L1 cognitive variables. L1 speech rate was controlled as an index of individual speaking style (the correlation between L1 and L2 speech rates was r = 0.68). The cognitive variables which correlated significantly across the languages (L1 rapid words and L1 Stroop repairs) were used as control variables, as these measures were closely related across the languages, and therefore, considered to tap into the same general cognitive process. The result from this analysis was saved as a residual to be used as the dependent variable in the second phase of regression analysis, with the L2 and cross-language cognitive measures that did not correlate across the language versions to tap into the more specific L2 measures (L2 interference, L2 facilitation, L2L1 interference, and L2L1 facilitation) as independent variables. The results can be seen in Table 4. After controlling for L1 speech rate and cognitive variables with high correlation to L2 counterparts, the L2 cognitive variables (the Stroop effects) explained some additional variance (6.7%) in the L2 speech rate, though this amount was not statistically significant.

**Table 4:** Regression analyses for L2 speech rate

	R Square	Adjusted R Square	Std. Error of the Estimate	R Square Change	F Change (df1, df2)	Sig. F Change
Step 1: L1 variables^a^	.487	.462	.347	.487	19,002 (3, 60)	< .001
Step 2: L2 variables^b^	.156	.067	.943	.156	1,757 (6, 57)	.124

^a^ Predictors: L1 corrections Stroop, L1 Rapid Words, Speech Rate L1 (per second)

Dependent Variable: Speech Rate L2 (per second)

^b^ Predictors: L2L1 Facilitation, L2 Facilitation, L1L2 Facilitation, L1L2 Inhibition, L2L1 Inhibition, L2 Inhibition

Dependent Variable: Standardized Residual

## Discussion

The aims of the present study were to examine the influence of both automaticity (Rapid words) and attention control (Stroop task) in lexical access on L2 speech rate (monologue in L2), while controlling for L1 speech rate (monologue in L1). Concerning RQ 1, on the connections between the L1 and L2 cognitive fluency measures, the correlation between the automaticity in recognising rapid words in L1 and L2 was strong for our advanced-level L2 participants. This correlation may be an indication of individual cognitive processing speed that applies to both L1 and L2 use, and fast word recognition is a more general cognitive trait (at least after a certain proficiency threshold, see Olkkonen et al., 2024). Notably, even with an extremely short exposure time (50 ms) and long, challenging words, the mean accuracy for L2 was 50%. As in priming tasks that have employed equally short exposure times (e.g., Zeguers et al., 2018), the participants had time to process what they had seen, but the reading process was limited by using a mask, thus ensuring that we could tap the automatic sight-word recognition rather than fast reading. These results highlight how efficiently this cohort of university students were able to lexically process even low-frequency words; this also warrants the question of how similar these results would be for less proficient or less educated cohorts, as the task relied heavily on breadth of vocabulary skills.

For the attention control in the Stroop task, the interference or facilitation effects did not correlate between the languages, which suggests that the effect is more dependent on the level of language skills. We found more interference from L1 on the reaction times, that is, from the more proficient language, as has been the case in many previous studies as well (e.g., Heidlmayr et al., 2014; MacLeod, 1991; Marian et al., 2013). This could make the strength of L2 interference a possible candidate for a language-specific index of cognitive processing (see, e.g., Kahng, 2020; Segalowitz, 2016). Concerning the number of repairs, an index of monitoring, it correlated significantly between L1 and L2. This correlation suggests that this may be more a personal trait or speaking style question, and that there may be some differences between the speakers regarding the functions and use of disfluencies, for example, between different task types (see, e.g., Peltonen, 2020). This is in line with the previous findings on the more conscious aspects of correcting and reformulating one’s speech (in speech: Zuniga & Simard, 2019; in Stroop: Shao et al., 2015), and is reflected also in different speaker profiles preferring either fast or accurate responses (Olkkonen et al., 2024). On the other hand, the finding of a larger number of repairs in L1 than in L2 lends further support to the hypothesis that disfluencies relating to monitoring are not always a result of low proficiency but, as in this case, due to more difficulty in inhibiting irrelevant information and/or more resources for spotting the errors.

In general, the results of the cognitive fluency measures were in line with previous studies, that, even for advanced learners, lexical access is less efficient in L2 compared to L1 (Kahng, 2014; Plat et al., 2018; Segalowitz, 2010; Towell & Dewaele, 2005) and the interference (i.e., automatic activation of information) is more pronounced from L1 than from L2 (Heidlmayr et al., 2014; MacLeod, 1991; Marian et al., 2013). Moreover, as could be evidenced from the large standard deviations, there were quite marked individual differences between the participants. The individuals differed, for example, on whether there was more interference *within* one language or always from L1 (the amount of intra-language and cross-linguistic interference), and the L2 profiles regarding the relationships of L2 speech rate, L2 proficiency, and cognitive fluency are quite different in advanced levels of proficiency (see Olkkonen et al., 2024).

Concerning RQ 2, as to the extent of variance in L2 speech rate explained by automatic and controlled lexical access, the findings were moderate. After controlling for L1 speech rate, which was responsible for the most variance explained (in line with previous studies, see, e.g., Kahng, 2020; Peltonen, 2020), and the L1 cognitive variables with high cross-language correlation, the L2 cognitive variables explained a small additional amount of variance (6.7%). The L2 cognitive fluency variables examined in this stage were L2 Stroop variables, and the finding that they managed to explain a unique, albeit small part of variance in L2 speech rate is notable, as this connection has not been established in previous studies. The large influence of L1 measures, however, is in line with the hypothesis (e.g., Duran-Karaoz & Tavakoli, 2020) that, for the advanced language users, the personal speaking or cognitive processing styles are quite audible in the L2 speech as well.

Next steps for the cognitive framework used here would be then to examine how they relate to other, more specific utterance fluency measures besides speech rate. In fact, specific effects in the Stroop task have been shown to connect to different types of repairs, most notably false starts (see Peltonen et al., 2024), and it is possible that the cognitive measures used here are especially relevant in helping to interpret the functions and occurrences of disfluencies. This more detailed understanding of the role of specific disfluencies in relation to cognitive processing is essential for a more comprehensive understanding of factors influencing L2 speaking performance. Furthermore, many current studies emphasise the location of pauses, instead of their number, as one of the most marked differences between L1 and L2 speech behaviour (e.g., Kahng, 2014, 2020). An interesting follow-up could be to study how the current cognitive processing resources framework could shed light on this, as pauses most likely occur in places where cognitive resources are stretched.

To sum up, the aim of the current paper was to study cognitive fluency variables in relation to speech rate as an index of speaking performance, by applying the general cognitive framework of automatic and controlled processing in a low-level task (lexical access). Automaticity in lexical access was found to be strongly related across the L1 and L2, whereas the difference between L1 and L2 attention control was quite marked even for the advanced L2 users studied here. In addition to automaticity, the strong correlation that was found between monitoring efficiency in L1 and L2 (number of repairs in the Stroop task) suggests that these measures were more related to general cognitive traits or preferences. This supports only partly the hypothesis that automaticity and control are in a complementary relationship, and points to a need for a more nuanced approach, for example, concerning different speaker profiles. As the number of participants was fairly low and the correlations moderate at best, more detailed analyses concerning especially different speaker profiles, as well as more fine-tuned examinations of the connections between specific aspects of cognitive and L2 utterance fluency, are obviously needed. Furthermore, the participants in the current study were very advanced learners, with knowledge of several additional languages besides L1 and L2, and therefore, examining how these results apply to different levels of proficiency is crucial in understanding the development and change in the cognitive profiles experienced by language learners. Nevertheless, the finding that, after controlling for L1 speech rate and cognitive fluency measures, the L2 cognitive fluency measures explained a small amount of additional variance in L2 speech rate offers tentative support for the framework used here, and, furthermore, to the hypothesis that these variables may work as possible candidates for L2 cognitive fluency that influence L2 fluency in general.

## Data Availability

After the research project has been concluded (2024), the data will be made partly available for other researchers through FIN-Clarin (the Language Bank of Finland). As parts of the research records contain directly identifiable data (the audio recordings), access to the data will be restricted (CLARIN RES license for restricted resources). The anonymised spreadsheet data that support the findings of this study are available from the corresponding author upon request.

## Footnotes

1 In addition, detailed analyses of various L1-L2 utterance fluency measures of the same data set are available (Peltonen et al., in prep.) focusing on the links between L1 and L2.
